# A Role for Central Nervous Growth Hormone-Releasing Hormone Signaling in the Consolidation of Declarative Memories

**DOI:** 10.1371/journal.pone.0023435

**Published:** 2011-08-05

**Authors:** Manfred Hallschmid, Ines Wilhelm, Christian Michel, Boris Perras, Jan Born

**Affiliations:** 1 Department of Neuroendocrinology, University of Lübeck, Lübeck, Germany; 2 Department of Internal Medicine I, University of Lübeck, Lübeck, Germany; 3 Department of Medical Psychology and Behavioral Neurobiology, University of Tübingen, Tübingen, Germany; University of Pennsylvania School of Medicine, United States of America

## Abstract

Contributions of somatotropic hormonal activity to memory functions in humans, which are suggested by clinical observations, have not been systematically examined. With previous experiments precluding a direct effect of systemic growth hormone (GH) on acute memory formation, we assessed the role of central nervous somatotropic signaling in declarative memory consolidation. We examined the effect of intranasally administered growth hormone releasing-hormone (GHRH; 600 µg) that has direct access to the brain and suppresses endogenous GHRH via an ultra-short negative feedback loop. Twelve healthy young men learned word-pair associates at 2030 h and were administered GHRH and placebo, respectively, at 2100 h. Retrieval was tested after 11 hours of wakefulness. Compared to placebo, intranasal GHRH blunted GH release within 3 hours after substance administration and reduced the number of correctly recalled word-pairs by ∼12% (both *P*<0.05). The impairment of declarative memory consolidation was directly correlated to diminished GH concentrations (*P*<0.05). Procedural memory consolidation as examined by the parallel assessment of finger sequence tapping performance was not affected by GHRH administration. Our findings indicate that intranasal GHRH, by counteracting endogenous GHRH release, impairs hippocampal memory processing. They provide first evidence for a critical contribution of central nervous somatotropic activity to hippocampus-dependent memory consolidation.

## Introduction

Studies in animals [1]–[3] and clinical observations in humans [4]–[7] suggest that somatotropic axis activity benefits memory function. However, little is known about the actual contribution of growth hormone (GH) and its hypothalamic secretagogue, growth-hormone releasing hormone (GHRH), to dissociable stages of memory formation in humans [8], [9]. Recent research has highlighted the role of neuroendocrine secretion patterns associated with sleep in the consolidation of memories [10]. Secretory activity of the somatotropic system shows a 24-hour maximum during the first night-half in close temporal association with episodes of slow-wave sleep that has been found to mediate improving effects on the consolidation of declarative, hippocampus-dependent memory contents (i.e., facts and episodes [11], [12]). Therefore, the release of GHRH and GH has been repeatedly suspected to actively contribute to processes of memory consolidation [13], [14]. However, blocking pituitary GH secretion in the first half of nocturnal sleep by intravenous infusion of somatostatin does not affect memory consolidation in healthy humans, precluding a direct influence of systemic GH on acute memory consolidation [15]. As circulating somatostatin does not cross the blood-brain barrier (BBB), this finding leaves open that effects of the somatotropic axis on declarative memory consolidation are conveyed by GHRH. Here, we investigated in healthy humans the effect of intranasal GHRH on declarative and procedural memory consolidation. The intranasal route of administration was chosen because it provides immediate access to the brain compartment [16]–[18].

## Methods

### Ethics statement

The study protocol conformed to the Declaration of Helsinki and was approved by the ethics committee of the University of Lübeck. All participants gave written informed consent.

### Subjects

We studied 12 healthy, right-handed young men aged 19 to 28 years (mean ± SEM: 23.3±1.0 years) with a mean body mass index between 20 and 25 kg/m^2^ (23.5±0.5 kg/m^2^). All subjects had a regular self-reported sleep-wake cycle during the last four weeks before the experiments as stated in pre-enrollment interviews and abstained from caffeine and alcohol intake on experimental days. Exclusion criteria were chronic or acute illness, current medication of any kind, smoking and drug abuse.

### Study design and procedure

Subjects participated in two experimental conditions, GHRH and placebo, according to a randomized and balanced cross-over design. In the GHRH condition, subjects were intranasally administered 600 µg GHRH_1–44_ (Bachem, Heidelberg, Germany) dissolved in 6 ml saline solution. In the placebo condition saline solution was administered. For administration, each subject received two 100 µl nasal atomizer puffs (one per nostril) every 1 min over a period of 30 min. Subjects and experimenter were blind with regard to the experimental condition. The two sessions of an individual subject were separated by at least two weeks.

On experimental days, subjects arrived at the lab at 1930 h. For blood sampling, a venous cannula was inserted into the subject's dominant arm. At 2030 h, subjects learned a list of word pairs for declarative memory testing and learned a procedural memory task (see below). From 2130–2200 h, GHRH and placebo were administered depending on condition. The timing of GHRH administration was chosen to cover the nocturnal period of enhanced somatotropic activity. In order to exclude interfering influences of sleep-related processes, subjects stayed awake throughout the night, watching a standardized set of non-exciting movies and playing non-verbal board games with the experimenter. Brisk physical activities were not allowed, and participants were constantly monitored by the experimenter to ensure wakefulness. They were allowed to drink water and were offered two light snacks at 0000 h and 0615 h to exclude confounding effects of increased hunger on cognitive measures. At 0900 h, i.e., after a post-treatment consolidation interval of 11 hours, recall of word pairs and procedural skill performance was tested. Heart rate and blood pressure were monitored twice in the evening and twice in the morning.

### Memory tasks

To investigate the effects of GHRH on the consolidation of declarative memory, a word pair interference paradigm was used previously shown to be highly sensitive to experimental manipulations of the consolidation process [19]. The task uses words drawn from a pool of nouns matched for imageability, frequency and concreteness that were randomly divided into three groups of 20 words, thus yielding three lists, A, B and C, with assignments to lists B or C counterbalanced across participants. Each word in the A list is paired with one word each from the B and C lists, thereby forming two lists of paired associates: A–B and A–C (e.g., winter-curd and winter-drawer). Parallel versions of the lists were used for the two test sessions. At 2030 h (learning phase), participants learned the first, i.e., A–B list of 20 word pairs. The word pairs were presented in capital letters on a computer screen for 4 s each. The whole list of word pairs was presented sequentially, in a fixed order across subjects and conditions. Immediately afterwards, participants were presented with the first (A) word of each pair and were required to type the corresponding (B) word. The computer provided immediate feedback by displaying the correct pairing for 4.5 seconds. Presentation of the list and immediate testing was repeated until subjects correctly recalled 90% of the words. After a post-treatment 11-hours retention period spent awake, subjects learned the new, interfering list of word pairs (A–C) according to the same training procedure as for the A–B list. After learning this new list, subjects performed a 15-min distractor task to prevent rehearsal between interference learning and final retrieval testing. At retrieval, subjects were presented the first (A) words and asked to recall the paired words from both lists, i.e., B and C of the A–B and A–C lists. If subjects only remembered one of the two completions, they were to leave the respective column blank. Orthographical errors were counted as correct. Only those words that were recalled and identified with the correct cue word (A) and placed in the correct list (B column if learned before the retention interval or C column if learned afterwards) were counted as accurate. (The number of correctly recalled words placed in an inappropriate list turned out to be negligible and was not further analysed.) Thus the paradigm measures the extent to which the memory for the A–B associations is weakened by interpolated learning of the interfering A–C associations. Accordingly, the primary outcome measure of consolidation was the difference between the number of correctly remembered A–B associations at retrieval and at learning.

Procedural memory was assessed using a finger sequence tapping task (adopted from [20]) requiring subjects to press four numeric keys on a standard computer keyboard with the fingers of their left (non-dominant) hand, repeating the five-element sequence as quickly and accurately as possible for a period of 30 seconds. The numeric sequence is displayed on the computer screen at all times to exclude any working memory component to the task. Each key press produces a white dot on the screen, forming a row from left to right, rather than the numbers typed so as not to provide accuracy feedback. Two motor patterns containing completely unique grammars (4-1-3-2-4 and 2-3-1-4-2) were used in a balanced order in the two experimental sessions. The training session took place at around 2100 h, i.e., after the study phase of the declarative memory task had ended, and consisted of twelve 30-sec trials with 30-sec rest periods between trials, lasting a total of 12 min. After the 11-hours post-treatment retention period and after word pair retrieval testing, performance on three trials of the finger sequence tapping task was probed. Each 30-sec trial was scored for the number of complete sequences achieved, designated as speed, and the number of correct sequences, designated as accuracy. The averaged scores (speed and accuracy) from the final three trials of the training sessions were taken as baseline measures and scores from the respective three 30-sec test trials were also averaged.

### Mood and vigilance assessment

Self-reported mood was rated on 5-point scales covering the categories good/bad mood, alertness/sleepiness and calmness/agitation (MDBF [21]) before learning, after substance administration and every 90 min thereafter with a final assessment taking place before retrieval testing (0900 h). In parallel, hunger and thirst were rated on 10-point scales. Sleepiness was assessed with the Stanford Sleepiness Scale and, on these occasions, subjects also performed a simple PC based vigilance task. In this task, a dot (10 mm diameter) appeared every 2, 4, 6, 8 or 10 seconds in the left or right field (randomly selected) of a computer screen and subjects were required to press corresponding left and right keys as fast as possible. Subjects received immediate feedback in the form of the reaction time (in ms) after a correct response or the signal ‘Wrong!’ after an incorrect response. The task included 40 trials (20 per screen half) and lasted approximately 5 min. For each 5-min task, mean reaction time, error rate and the number of lapses (reaction time >500 msec) were registered.

### Blood sampling and assays

Blood was sampled for the determination of GH, adrenocorticotropin (ACTH), cortisol, catecholamines, blood glucose and insulin before substance administration (2025 and 2125 h) and immediately thereafter at 2205 h. From 2215 h on, samples were taken every 20 min until 2315 h, every 30 min until 0045 h and every 60 min until 0845 h. A final sample was collected after retrieval testing. Blood glucose was determined immediately after sampling (HemoCue B-Glucose-Analyzer, Ängelholm, Sweden). The remaining samples were centrifuged and serum and plasma were frozen at −80°C for later analysis. Serum GH, cortisol and insulin concentrations as well as plasma ACTH levels were measured by commercial enzyme-linked immunoassays (all Immulite, DPC, Los Angeles, CA, USA) with the following intra-assay and inter-assay coefficients of variation (CV) and limits of sensitivity (LOS), respectively: GH: <5.8% and <5.5%, LOS 0.05 µmol/l; cortisol: <5.8% and <6.3%, LOS 5.518 mmol/l; insulin: <5.2% and <6.1%, LOS 12.0 pmol/l; ACTH: <6.1% and <9.4%, LOS 0.4404 pmol/l. Plasma epinephrine and norepinephrine were measured by standard high-performance liquid chromatography with electrochemical detection (Chromosystems, Munich, Germany). Intra-assay and inter-assay CV was <2.9% and <4.2%, LOS 54.58 pmol/l for epinephrine and <2.6% and <3.9%, LOS 59.11 pmol/l for norepinephrine.

### Statistical analyses

Values are expressed as means ± SEM. Analyses were generally based on analyses of variance (ANOVA) for repeated measures, including the factors ‘treatment’ (for ‘GHRH’ vs. ‘placebo’) and ‘time’ (for repeated measurements during the session). Degrees of freedom were corrected according to the Greenhouse-Geisser procedure where appropriate. Single-time points comparisons using two-tailed paired t-tests were calculated in case ANOVA yielded significant treatment effects. A *P*-value less than 0.05 was considered significant.

## Results

### Intranasal GHRH impairs declarative but not procedural memory consolidation

**Figure 1:**
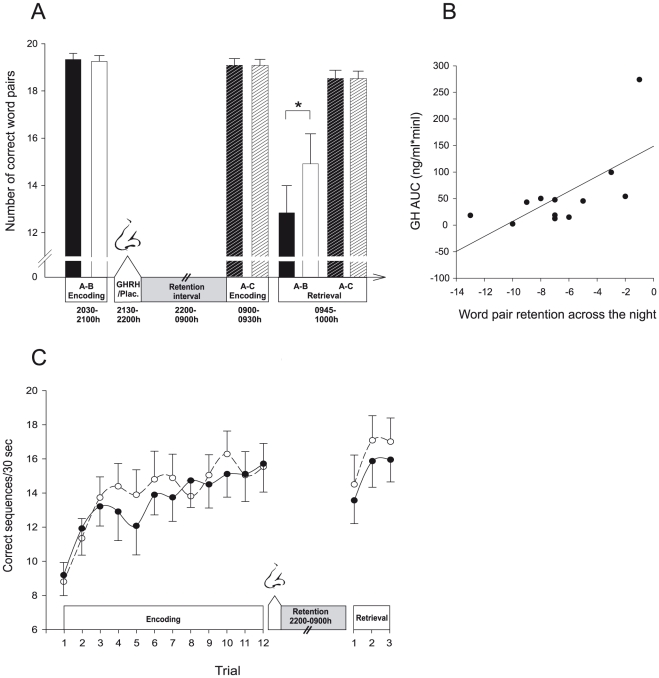
GHRH impairs declarative memory consolidation. (A) Declarative memory: Mean (± SEM) performance on the word pair interference task as assessed by the number of correctly recalled word pairs in the GHRH condition (black bars; intranasal administration of 600 µg GHRH_1–44_ from 2130–2200 h) and the placebo condition (white bars; vehicle). Encoding of the A–B word pair list (plain bars) took place before substance administration and a nocturnal retention interval of 11 hours spent awake. Encoding of the A–C word pair list (hatched bars) took place after the retention interval. Retrieval of both lists was jointly tested at the end of the session. GHRH administration significantly reduced retention of A–B word pairs whereas retrieval of the A–C interference list remained unaffected. * *P*<0.05. (B) Retention of word pairs in the GHRH condition plotted against respective 2200-0045 h plasma GH AUC values, indicating a positive correlation between consolidation of word pairs and post-learning somatotropic activity (r = 0.66, *P* = 0.019; note that excluding the subject with the highest plasma GH AUC value from analysis yielded essentially the same result, r = 0.61, *P* = 0.046). (C) Procedural memory: Mean (± SEM) finger tapping performance indicated by the number of correct sequences within a 30 sec trial during encoding and after the retention interval following intranasal administration of GHRH (black dots, solid lines) and vehicle (white dots, dashed lines), respectively. Encoding consisted of twelve 30-sec trials and retrieval testing comprised three 30-sec trials. Unlike declarative word pair memories, procedural finger tapping skill was not affected by GHRH. N = 12.

Learning performance on the declarative memory task, i.e., word pair learning, was comparable between both learning sessions (A–B list before the retention period and A–C interference list before retrieval) and between conditions in terms of the number of correct word pairs recalled during the criterion trial (Figure 1A; GHRH vs. placebo, A–B: 19.33±0.26 vs. 19.17±0.24; A–C: 19.08±0.26 vs. 19.09±0.25; all *P*>0.49) and of the number of trials needed to reach the criterion of 90% correctly recalled word pairs (A–B: 1.75±0.13 vs. 2.00±0.17; A–C: 2.00±0.17 and 1.91±0.21; all *P*>0.19).

Compared to the learning phase before the 11-hours retention period, at the final retrieval test in the morning subjects remembered distinctly less word pairs of the A–B list in both conditions, reflecting in part the weakening effect on these memories of interpolated learning of the A–C interference list (F(1,11) = 31.24, *P*<0.001 for Time). However, forgetting of A–B associations was more pronounced after GHRH administration (F(1,11) = 5.47, *P* = 0.039 for Treatment×Time). Accordingly, the difference in correctly recalled word pairs from the A–B list between learning and retrieval was greater in the GHRH condition (Figure 1A; GHRH vs. placebo, −6.5±1.16 vs. −4.25±0.99 word pairs, t = 2.34, *P* = 0.039; 65.92±5.4% and 77.45±6.24% of word pairs recalled at learning, t = 2.21, *P* = 0.05). In contrast to the A–B list, retrieval of the A–C interference list did not differ from learning performance and was also comparable between both conditions (*P*>0.15 for all comparisons; Figure 1A). Notably, in the GHRH condition, retention of the A–B list (as indicated by the absolute difference in correctly recalled word pairs between learning and retrieval) was significantly correlated with plasma GH concentrations as assessed by the area under the curve (AUC) 2200-0045 h (r = 0.66, *P* = 0.019; Figure 1B). This correlation was not significant in the placebo condition (r = 0.36, *P* = 0.244).

Finger sequence tapping performance was assessed with regard to accuracy (number of correct sequences per trial) and speed (number of sequences per trial). As the pattern of results was similar for both measures, only accuracy measures are reported here. During encoding before GHRH administration, task performance significantly increased (Figure 1C; mean number of sequences in the first three trials of encoding, GHRH vs. placebo, 11.19±1.33 vs. 11.36±1.08; in the last three trials, 15.53±1.61 vs. 15.75±1.31; F(1,11) = 44.48, *P*<0.0001 for Time) with no differences between conditions (F(1,11) = 0.06, *P* = 0.80 for Treatment, (F(1,11) = 0.006, *P* = 0.80 for Treatment×Time). Finger tapping performance did not change across the 11-h retention interval and was also not influenced by GHRH treatment (GHRH vs. placebo, 1.05±0.63 vs. −0.5±0.91 sequences, F(1,11) = 0.24, *P* = 0.63 for Time, F(1,11) = 2.12, *P* = 0.17 for Treatment×Time).

### GHRH does not affect vigilance and tiredness while improving subjective well-being

**Figure 2:**
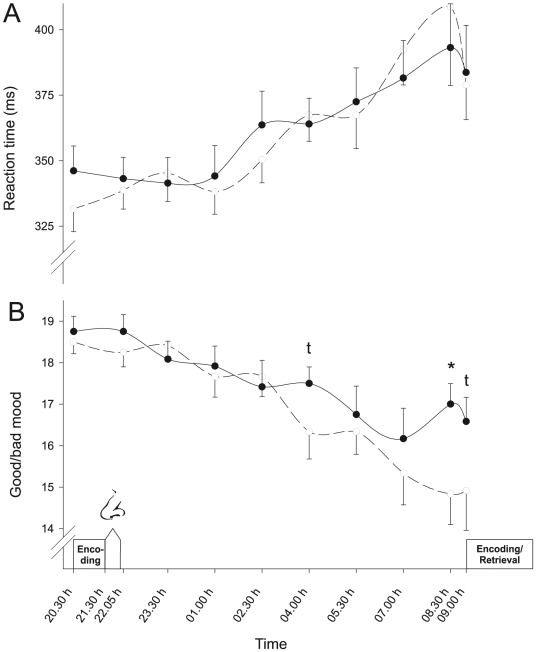
Vigilance and mood after GHRH and placebo administration. Mean (± SEM) (A) reaction times in a PC based vigilance task and (B) ratings of subjective well-being as derived from the ‘good/bad mood’ subscale of the MDBF questionnaire (with high values indicating good mood [21]) during the nocturnal retention period spent awake following intranasal administration of 600 µg GHRH_1–44_ (black dots, solid lines) and vehicle (white dots, dashed lines), respectively. Periods of substance administration and encoding/retrieval of the memory tasks are indicated. N = 12,^t^ p<0.10, * *P*<0.05.

Subjects' performance on the vigilance test deteriorated during the night (*P*<0.01, for Time effects regarding reaction time, errors and lapses), but was not affected by treatment (*P*>0.55 for Treatment and Treatment×Time effects; Figure 2A). In accordance, sleepiness assessed by the Stanford Sleepiness Scale increased from baseline values at 2030 h (GHRH vs. placebo, 1.83±0.21 vs. 1.75±0.22) to reach maximum values at 0830 h (4.83±0.30 vs. 5.42±0.27, *P*<0.001) but was not affected by GHRH treatment (all *P*>0.54). Rated hunger and thirst were likewise unaffected by GHRH administration (*P*>0.46, for all comparisons). Subjective well-being as assessed by the MDBF subscale ‘good/bad mood’ deteriorated during the night (*P*<0.001). GHRH treatment attenuated this decrease and improved mood in comparison to placebo values (F(3,35) = 3.39, *P*<0.03 for Treatment×Time), yielding significantly higher values towards the end of the session (Figure 2B). Mood improvement after intranasal GHRH was not related to the deterioration of declarative memory consolidation in this condition (*P*>0.15 for all correlations between mood ratings at 0830 h and 0900 h and measures of memory retrieval).

### Intranasal GHRH reduces GH secretion

**Figure 3:**
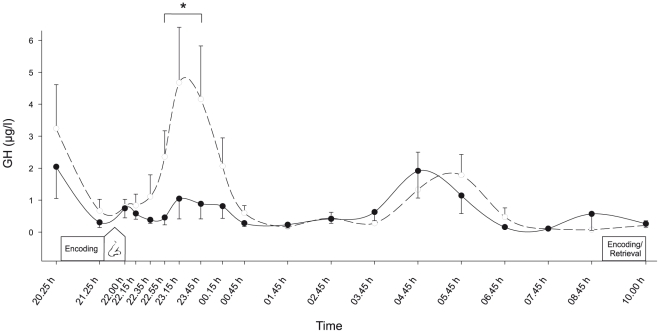
Intranasal GHRH decreases circulating GH concentrations. Mean (± SEM) serum concentrations of GH during nocturnal wakefulness following intranasal administration of 600 µg GHRH_1–44_ (black dots, solid line) and placebo (white dots, dashed line), respectively. Administration of GHRH and placebo took place after encoding of declarative and procedural memory tasks that were retrieved 11 hours later after encoding of an interference word pair list. N = 12, * *P*<0.05.

During baseline before substance administration, circulating hormone concentrations and blood glucose levels were closely comparable between conditions (all *P*>0.38). Circulating GH concentrations that showed a small but distinct GH peak in the control condition during the first night half were markedly reduced by intranasal GHRH administration (Figure 3; F(1,11) = 10.18, *P*<0.009 for Treatment, *P* = 0.16 for Treatment×Time). Accordingly, area under the curve (AUC) analyses covering the first three hours after GHRH administration, i.e., a time interval when based on previous observations maximal treatment effects were to be expected [12], [16], [22], indicated distinctly blunted GH plasma concentrations following GHRH as compared to placebo administration (56.70±21.15 vs. 201.04±65.12 µg/l*min, *P* = 0.046). Likewise, individual GH peak values reached during this time period were markedly smaller in the GHRH condition (1.89±0.67 vs. 6.57±1.88 µg/l, *P* = 0.022). Thereafter, GH concentrations did not differ between conditions.

Blood glucose concentrations (*P*>0.31 for respective Treatment and Treatment×Time effects) as well as circulating concentrations of insulin (*P*>0.22), ACTH (*P*>0.15), cortisol (*P*>0.70), epinephrine (*P*>0.36) and norepinephrine (*P*>0.54) were not affected by treatment. Across conditions, ACTH and cortisol showed the expected circadian increase in the second night half (both *P*<0.001). Heart rate and blood pressure remained unaffected by GHRH treatment (all *P*>0.22). Interviews at the end of the sessions confirmed that subjects could not correctly indicate if they had received placebo or GHRH (*P* = 0.41, chi-square test).

## Discussion

GHRH administered intranasally following the acquisition of two memory tasks impaired consolidation of declarative but not procedural memory. GHRH also decreased plasma GH concentrations between 1–2 hours post-administration as reflected by the complete blunting of a peak in GH release emerging during the nocturnal retention period in the placebo condition. In combination, both effects support the view that inhibiting somatotropic activity at the central nervous level impairs the acute consolidation of declarative, i.e., hippocampus-dependent memory traces.

The inhibition of GH secretion from the pituitary by intranasal GHRH administration is seemingly paradoxical. However, it replicates previous results indicating that intranasal GHRH reduces the strong surge in GH release that is normally associated with early nocturnal sleep [22]. Here, because our subjects were awake to exclude interfering influences of sleep-related processes, the early nocturnal GH peak was distinctly smaller [23], [24]. Nevertheless, it was entirely blunted by intranasal GHRH. In rats, the intracerebroventricular injection of low doses of GHRH likewise reduces pituitary GH release [25]. The suppressing effect of intranasal GHRH on pituitary GH secretion can thus be explained by exogenous GHRH activating an ultra-short negative feedback mechanism on endogenous, hypothalamic GHRH secretion [26]. According to in vitro-studies, this feedback-suppression of somatotropic activity may involve activation of somatostatin releasing neurons [27]. On the background of reduced instead of enhanced pituitary GH secretion, peripheral effects of intranasal GHRH can be safely excluded. Also, previous studies have demonstrated unchanged GHRH levels in plasma after intranasal administration of the compound [28].

The central finding of our study indicates impaired consolidation of hippocampus-dependent declarative memory by intranasal GHRH administered after the learning period. GHRH administration made the word pair associations acquired before substance administration more vulnerable to the disturbing influence of associative interferences learned shortly before retrieval testing. A persisting effect of GHRH immediately acting on retrieval function can be excluded because immediate recall of the interfering word list newly acquired at the time of the final retrieval test was completely unaffected. Experiments were conducted during nocturnal wakefulness to differentiate purely somatotropic from sleep-related influences on memory consolidation, which, on the other hand, limits the transfer of our results to the regular sleep setting. GHRH administered via the intravenous [29] but also the intranasal route [22] has moderate sleep-enhancing properties, mainly on slow wave sleep [26], [30]. Our finding of comparable vigilance and sleepiness measures in both conditions is consistent with the observation that any sleep-promoting effect of GHRH is particularly small at times of high sleep propensity (i.e., late evening [31]). On the other hand, it excludes a biasing influence of sleepiness on recall performance. Also, improved mood in the GHRH as compared with the placebo condition was unrelated to the deterioration of memory consolidation due to GHRH administration.

The impairing influence of intranasal GHRH was specific to the consolidation of declarative word pairs. Procedural finger sequence tapping performance in both conditions reached a post-training level lower than in some [32], [33] but not all comparable previous studies [34], [35] where tapping performance nevertheless showed the typical sleep-dependent improvement. In contrast to word pair consolidation, results of the finger sequence task remained completely unaffected by GHRH administration. Processing of declarative memory requires the involvement of hippocampal structures [36], [37]. Endogenous somatotropic activity reaches a maximum during the early night-half in close temporal association with the first periods of nocturnal slow wave sleep that has been demonstrated to support preferentially the consolidation of hippocampus-dependent memory [12], [38], [39]. Against the background of the well-known slow wave sleep-promoting properties of GHRH administration [26], [30], those findings stimulated the assumption that somatotropic axis activity facilitates memory formation [13], [14]. In accordance, clinical studies on the subchronic administration of GH and GHRH in GH-deficient humans and in healthy older adults have revealed some evidence that somatotropic hormones are involved in the long-term maintenance of memory systems [4]–[7]. Recent animal data indicate that repetitive injections of GH can alleviate the impairing effects of prolonged sleep deprivation on hippocampal N-methyl-D-aspartate (NMDA) glutamate receptor function [2]. However, up to now there has only been one study systematically investigating the effects of somatotropic activity on the retention of specific memory contents [15]. In that study, post-learning inhibition of pituitary GH release by somatostatin infusion abrogated the early nocturnal GH peak but failed to affect sleep-associated consolidation of memory for word pairs learned in the preceding evening. Importantly, to reduce GH secretion, intravenous somatostatin acts on the pituitary that is located outside of the BBB, but it does not pass the BBB [40], [41]. Thus, any significant contribution of peripheral GH to sleep-related declarative memory processing was ruled out. In conjunction with those findings, the present data identify brain-borne GHRH as the factor conveying the influence of somatotropic axis activity on the consolidation of hippocampus-dependent memories: The inhibition of somatotropic activity at the central nervous level, via activation of ultra-short negative feedback induced by intranasal GHRH, but not by somatostatin acting at the pituitary, impairs declarative memory consolidation. This assumption is further buttressed by the significant correlation between declarative memory retrieval and circulating GH levels, which directly reflect central nervous GHRH secretion, that emerged in the GHRH condition and suggests that somatotropic and cognitive treatment effects were strongly linked. In the placebo condition, saturation due to higher central nervous GHRH levels might have prevented that slight variations in GHRH levels manifested themselves in corresponding variations in memory performance.

One plausible scenario accounting for the central nervous memory effects of GHRH assumes a direct action of the peptide on memory related brain structures. GHRH is predominantly produced in the arcuate nucleus of the hypothalamus with projections to the median eminence. Projections between hypothalamic and hippocampal structures are well documented [42], [43] and GHRH has been traced in extrahypothalamic regions including amygdala, hippocampus and cortical regions [44]–[47] where it probably acts as a neurotransmitter and/or neuromodulator [45], [48]. Interestingly, ghrelin that stimulates pituitary GH via GHRH-dependent pathways [49] and shares receptor mechanisms with GHRH [50] has been shown to bind to hippocampal neurons, promoting dendritic spine synapse density and spatial memory [51], [52], which tempts to speculate that GHRH exerts comparable hippocampal effects. In addition, endogenous GHRH might improve memory processing by enhancing central nervous GH and IGF-1 signaling [53], [54] that can promote declarative memory by inducing gene expression for hippocampal glutamatergic NMDA receptors [1], [3], [53], [55].

In sum, this is the first study to show that central nervous GHRH is involved in the consolidation of declarative memories by demonstrating that intranasal GHRH administration, by a presumptive immediate negative feedback action on central nervous GHRH networks, makes declarative memory contents more vulnerable to interfering influences. Our findings support the notion that somatotropic axis activity, which is most prominent during early sleep, contributes to the consolidation of hippocampus-dependent memories by enhanced GHRH feed forward signaling.
